# Sustainable Plant Growth Promotion and Chemical Composition of Pyroligneous Acid When Applied with Biochar as a Soil Amendment

**DOI:** 10.3390/molecules27113397

**Published:** 2022-05-25

**Authors:** Keiji Jindo, Travis Luc Goron, Soboda Kurebito, Kazuhiro Matsumoto, Tsugiyuki Masunaga, Kouki Mori, Kazuhiro Miyakawa, Seiya Nagao, Takeo Tokunari

**Affiliations:** 1 Agrosystems Research, Wageningen University & Research, P.O. Box 16, 6700 AA Wageningen, The Netherlands; 2 Department of Plant Agriculture, University of Guelph, Guelph, ON N1G 2W1, Canada; gorontr@uoguelph.ca; 3 Meiwa Co., Ltd., 3-8-1 Minato, Kanazawa 920-0211, Japan; b-so@meiwa-ind.co.jp (S.K.); okunari@mde.harvard.edu (T.T.); 4 Faculty of Agriculture, Shizuoka University, 836 Ohya, Suruga-ku, Shizuoka 422-8529, Japan; matsumoto.kazuhiro@shizuoka.ac.jp; 5 Faculty of Life and Environmental Sciences, Shimane University, 1060 Nishikawatsu, Matsue 690-8504, Japan; masunaga@life.shimane-u.ac.jp (T.M.); orest618365@gmail.com (K.M.); 6 Graduate School of Natural Sciences and Technology, Kanazawa University, Kanazawa 920-1192, Japan; maythird1@gmail.com; 7 Low Level Radioactivity Laboratory, Institute of Nature and Environmental Technology, Kanazawa University, Kanazawa 920-1192, Japan; seiya-nagao@se.kanazawa-u.ac.jp; 8 Harvard John A. Paulson School of Engineering and Applied Sciences, Science and Engineering Complex, 150 Western Avenue, Boston, MA 02134, USA; 9 Harvard Graduate School of Design, 48 Quincy Street, Cambridge, MA 02138, USA

**Keywords:** pyroligneous acid, pyrolysis, biostimulant, biorefinery, bio-oil, pyrolysis liquid

## Abstract

The pyrolysis of biomass material results in pyroligneous acid (PA) and biochar, among other by-products. In agriculture, PA is recognized as an antimicrobial agent, bio-insecticide, and bio-herbicide due to antioxidant activity provided by a variety of constituent materials. Application of PA to crop plants and soil can result in growth promotion, improved soil health, and reduced reliance on polluting chemical crop inputs. More detailed information regarding chemical compound content within PA and identification of optimal chemical profiles for growth promotion in different crop species is essential for application to yield effective results. Additionally, biochar and PA are often applied in tandem for increased agricultural benefits, but little is known regarding the optimal proportion of each crop input. This work reports on the effect of combined applications of different proportions of PA (200- and 800-fold dilutions) and chemical fertilizer rates (100%, 75%, 50%, and 0%) in the presence or absence of biochar on Komatsuna (*Brassica rapa* var. *perviridis*, Japanese mustard spinach) plant growth. To elucidate the chemical composition of the applied PA, four different spectroscopic measurements of fluorescence excitation were utilized for analysis—excitation-emission matrix, ion chromatography, high-performance liquid chromatography, and gas chromatography-mass spectrometry. It was determined that PA originating from pyrolysis of Japanese pine wood contained different classes of biostimulants (e.g., tryptophan, humic acid, and fulvic acid), and application to Komatsuna plants resulted in increased growth when applied alone, and in different combinations with the other two inputs. Additionally, application of biochar and PA at the higher dilution rate increased leaf accumulation of nutrients, calcium, and phosphorus. These effects reveal that PA and biochar are promising materials for sustainable crop production.

## 1. Introduction

Application of biochar in agricultural and environmental contexts has increased recently, partially due to advantages in terms of mitigating climate change, carbon sequestration, soil fertility (increased water holding capacity, nutrient retention, bulk density), and remediation of air, soil, and water. Biochar is produced by pyrolysis—the thermal degradation and carbonization of a carbonaceous material in the absence of oxygen. In addition to biochar, other materials are generated, including pyroligneous acid (PA), tar, and syngas (a fuel mixture of hydrogen, carbon monoxide, and often, some carbon dioxide).

PA (also referred to as wood vinegar, pyroligneous liquor, pyroligneous extract, pyrolysis oil, or aqueous phase bio-oil) is a liquid material with condensed and highly oxygenated organic acids, arising from reactions between volatile elements generated during thermal decomposition. PA is composed of water (80–90%) and more than 200 organic compounds, including acids, alcohols, phenols, aldehydes, and esters (10–20%) depending on pyrolysis conditions [1,2,3,4,5]. Some of these compounds have antioxidant properties and can be developed into a range of useful products [6,7,8,9,10].

Due to this antioxidant activity, in agriculture, PA is recognized as a valuable antimicrobial agent and bio-insecticide. For example, the high concentration of phenol compounds in PA can enhance plant protection mechanisms [11]. In addition, PA contains other chemical compounds which may act as biodegradable herbicides, including 5-aminolevulinic acid (ALA) [12].

Additional benefits of PA for agriculture include promotion of plant growth [1] and soil health [13,14]. Application of PA has been observed to increase seed germination and root growth [15], perhaps the result of organic acids converting unavailable soil P into phosphoric acid for nutrient uptake [16]. According to Wang et al. [17], several plant-growth promoting mechanisms may be triggered in parallel by PA application: (1) accumulation of proteins involved in different pathways of secondary metabolism, stress response, and carbohydrate metabolism; (2) accumulation of antioxidant enzymes, and (3) decreased reactive oxygen species (ROS) and malonaldehydes in root tissue [17]. These pathways are essential for proper molecular regulation of plant growth in response to stress.

However, knowledge regarding PA application in agriculture is mostly derived from limited studies focusing on plant growth effects. Detailed information regarding chemical compound content within PA and identification of optimal chemical profiles for growth promotion in different crop species is essential for application to be effective. For example, PA is reported to contain humic substance (complex heterogeneous organic compounds) mixtures, consisting of fulvic and humic acids. Humic substances are well-known plant-growth biostimulants [18], but more research is needed. It is also important to characterize exact chemical composition as PA is often assumed to be a biodegradable material [19], with little concern for environmental effects following application. Spectroscopic techniques may be used for better characterization of PA chemical composition and effects on plant growth [20].

Another challenge is to define optimal PA concentrations for specific crops. For example, Zulkaramai et al. [21] compared the effect of four concentrations of PA (0, 10, 20, and 30%) and found that although 30% was toxic to rockmelon (*Cucumis melo* L. *cv*.), a concentration of 10% was beneficial for plant growth.

Finally, biochar and PA are often applied in tandem for various uses, such as enhancing seed germination and growth [22], enhancement of protection against microbial activity [23], and the degradation of herbicide [24]. Previously, Pan et al. [25] hypothesized that positive effects of combined application might be due to several factors, including increased soil fertility and nutrient supply. It is therefore important to monitor changes in soil nutrients after the addition of PA and/or biochar.

This work reports on the effect of combined applications of chemical fertilizer, biochar, and PA on plant growth in a pot experiment (treatments described in Table 1). To characterize chemical profiles of the organic inputs, four spectroscopic measurements of fluorescence excitation were utilized—excitation-emission matrix (EEM) [5,10], ion chromatography [26], high-performance liquid chromatography (HPLC) [20], and gas chromatography-mass spectrometry (GC-MS) [8,26,27]. The effect of inputs on growth of Komatsuna plants (*Brassica rapa* var. *perviridis*, Japanese mustard spinach) was examined with three factors: (1) Two dilution rates of PA (200-fold and 800-fold); (2) Four levels of chemical fertilizer rates (100%, 75%, 50%, and 0%), and (3) biochar addition. This work aims to evaluate the effect of PA application on growth of *Komatsuna* in combination with biochar.

## 2. Results and Discussion

### 2.1. Chemical Analysis of PA

#### 2.1.1. EEM and SEC-HPLC

**Figure 1:**
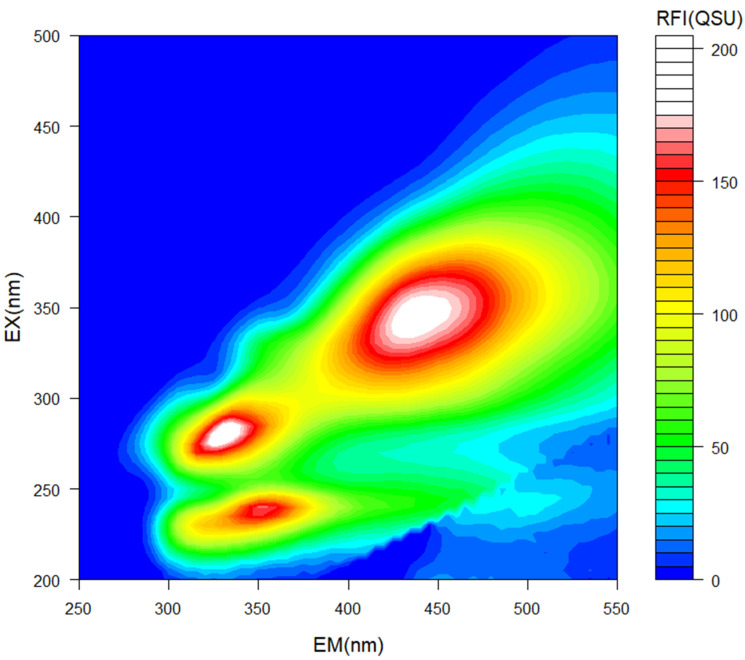
Excitation Emission Matrix (EEM) spectrometric analysis for identification of chemical compounds in pyroligneous acid. Emission (EM, x-axis) and excitation (EX, y-axis) spectra are represented in nanometers (nm). Color represents relative fluorescence intensity (RFI) in quinine sulfate units (QSU).

Excitation emission matrix (EEM) spectroscopy enables detection of compounds via fluorescence emission from organic substances. Three distinct characteristic regions were detected in our study, following EEM spectroscopy (Figure 1):

(1) E_x_/E_m_ = 230 nm/350 nm indicated the presence of a protein [28,29], likely a protein rich in tryptophan [30,31].
(2) E_x_/E_m_ =280 nm/335 nm indicated the presence of protein-like aromatic amino acids [32].
(3) E_x_/E_m_ = 340/430–450 indicated the presence of humic-like substances [33].

Tryptophan is a well-known biostimulant, which can promote plant growth by enhancing nutrient uptake [34]. Tyrosine and phenylalanine are aromatic amino acids, also considered to be biostimulant materials [35]. Humic-like substances have been previously identified in PA by other authors [5], and are often considered biostimulants for plant growth [36]. Humic-like substances may induce ATPase pumps in root tissues, thereby promoting root elongation through an auxin-like effect [36].

**Figure 2:**
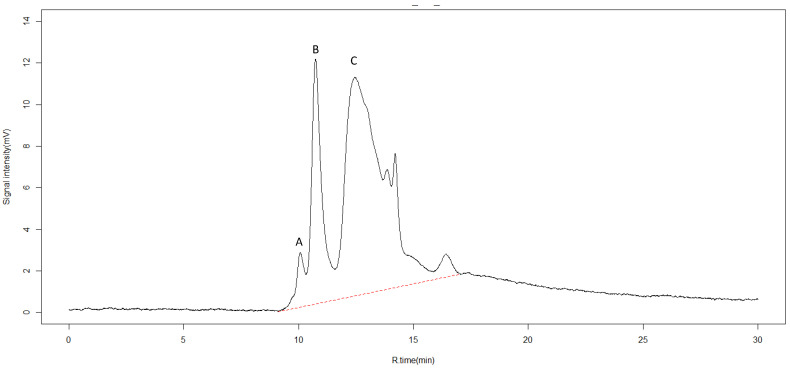
High pressure size exclusion chromatography (SEC-HPLC) for identification of chemical compounds in pyroligneous acid. X-axis represents retention time in minutes (R.time (min)). Y-axis represents intensity of absorbance in millivolts (mV). The dotted red line represents the range between the first and last detectable peak. Peaks observed at retention rates of 10.1, 10.7, and 12.4 min, labeled A, B, and C, were identified as humic and fulvic acids.

Organic compounds can also be characterized with SEC-HPLC [37,38]. Six different peaks were detected (Figure 2). The first three peaks observed at retention rates of 10.1, 10.7, and 12.4 min were identified as organic complexes of humic acid and fulvic acid, as reported in other works [39]. Fulvic acid is considered a biostimulant material [40] and increases plant root growth by inducing nodulation gene signaling, and stimulating beneficial bacteria such as *Rhizobium* [41]. The latter three peaks could not be identified.

#### 2.1.2. Ion Chromatography and GC-MS

Ion chromatography is often used to quantify organic acids within liquid samples. Acetic acid was the major component within the PA examined in this study, making up 41% of the total PA (4100 mg 100 mL^−1^) and 73.8% of all detectible oxide acids (Table 2). This range is congruent with other studies—acetic acid has been found by other authors to account for 80% of detectable compounds in PA [12,26]. In order of decreasing concentrations, pyruvic acid (13%; 1300 mg 100 mL^−1^), succinic acid (1.1%; 110 mg 100 mL^−1^), and malic acid (0.4%; 40 mg 100 mL^−1^) were also detected.

The quantity of compounds within PA is largely affected by pyrolysis temperature. Compared to temperatures used in other studies, the pyrolysis temperature used here was high (400 to 500 °C vs. 150 to 270 °C [42]). Lower temperatures have been noted to produce PA containing higher concentrations of acetic acid [43]. 

GC-MS is also widely used to characterize the composition of PA products [44,45]. Quantitative measurement of detected compounds can be performed by comparison of areas underneath an identified chromatographic peak. The following phenolic and aromatic compounds were identified by GC-MS: acetic acid, 2-hydroxyethyl acetate, cyclopentanone, O-guaiacol, vanillin, 2-methoxy 4 methylphenol (creosol), 2′-hydroxy-5′-methoxyacetophenone (acetophenone), and levoglocosan (Table 3). Creosol is not only often present in liquid smoke [26], but also a well-known disinfectant and antiseptic [42].

### 2.2. Field Trial

Komatsuna (*Brassica rapa* var. *perviridis*) plants were grown in greenhouse pots receiving different dilution rates of PA, biochar, and chemical fertilizer (Table 1). Fresh weights were recorded (Table 4). Following individual application of biochar and chemical fertilizer, significant differences were observed. Significant interaction was detected between biochar addition and chemical fertilizer. The interaction between all three factors (PA, biochar, and chemical fertilizer) was also significant. 

Highest values were generally observed in the 100% chemical fertilizer treatment. Of the other treatments, the multiple application of biochar, 200-fold PA, and chemical fertilizer (100%) was observed to produce the largest fresh weights. 

Interestingly, the effect of PA application alone at both dilution rates (800-fold and 200-fold) on fresh weight was significant (*p* < 0.05) when neither fertilizer nor biochar was applied. Under abiotic conditions, PA application may trigger enhancement of plant defense mechanisms for stress mitigation via production of reactive oxygen species involved in secondary metabolism [17]. 

The application of 800-fold PA produced slightly more total fresh biomass than 200-fold PA. Other studies agreed with this result, which indicates that high concentrations of PA (0.5 and 1.0 mL L^−1^) may negatively impact plant growth, compared to lower concentrations (0.25 mL L^−1^) [46]. Biochar application alone did not have a significant impact on biomass in this study. 

In terms of plant height (Table 5), application of different chemical fertilizer rates (50, 75, and 100%), when applied alone, were not significant. Inclusion of biochar also did not have a significant effect, except when paired with the 100% chemical fertilizer and PA treatment at 800-fold.

The effect of PA alone at either dilution rate with no fertilizer exceeded the control in plant height, similar to plant fresh weight. The impact of PA application on plant growth may become more apparent under extremely stressful conditions such as nutrient deficiency. No significant differences were observed amongst individual application of biochar to soil. 

**Figure 3:**
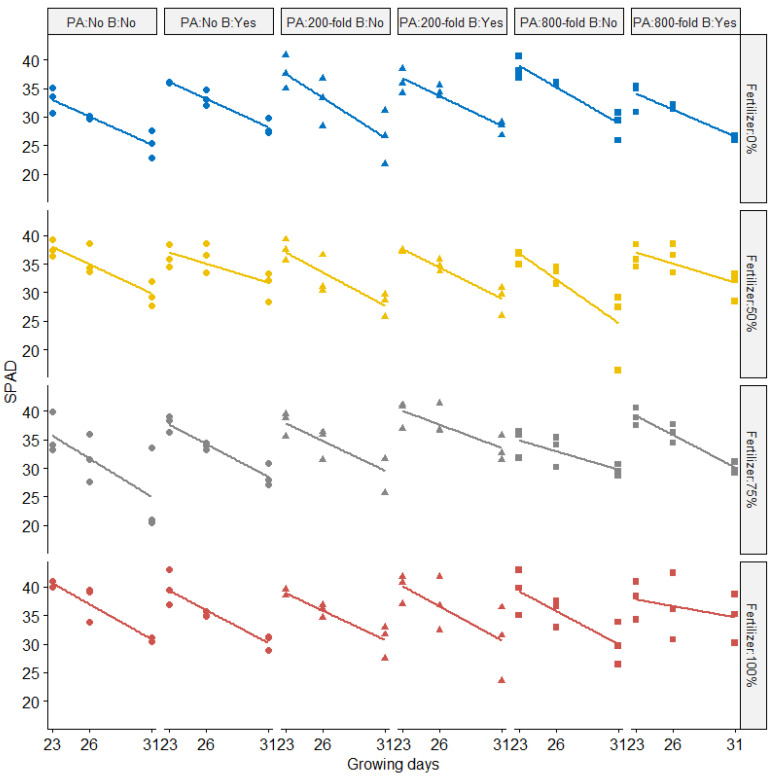
SPAD values from Komatsuna plants over three growing days, with fold dilution treatments of pyroligneous acid (PA), biochar (B), and chemical fertilizer rates of 0%, 50%, 75%, or 100%. Symbols represent the amount of pyroligneous acid (Circle: No application; Triangle: pyroligneous acid with 200-folddilution; Square: pyroligneous acid with 800-fold dilution while different colors represent the rates of chemical fertilizer (Blue: Fertilizer 0%; Yellow: Fertilizer 50%; Gray: Fertilizer 75%; Red: Fertilizer 100%).

SPAD (Soil Plant Analysis: Development; SPAD-502, Konica Minolta, Osaka, Japan) values were recorded over three different growing days (Figure 3). SPAD values were negatively correlated with growing days (*p* < 0.05) across all treatments. The amount of fertilizer applied resulted in significantly different SPAD values. On the final measurement day, most plants supplied with 100% of chemical fertilizer did not have drastically lower SPAD values compared to the 50% chemical fertilizer treatment. Significant effects on SPAD values following biochar addition (*p* < 0.05) were also observed across different treatments. No impact of PA dilution rate on SPAD was observed. Wang et al. [17] reported that PA can positively affect the root proteome and consequently delay plant senescence under laboratory conditions. However, the effect of PA in our study conducted under greenhouse conditions revealed a different result. Other studies [15] reported results similar to our experiments, showing little difference between control and PA-treated rapeseed plants over different development stages (seedling, bud bolting, and flowering) except for at the pod stage (182 days after sowing). Interactive effects between chemical fertilizer and biochar application on SPAD were observed in our study.

### 2.3. Effect on Plant Nutriton and Soil Properties

Regarding plant leaf nutrients, no significant differences were observed in K, Na, Fe, or Zn concentrations (Table 6). However, significant differences (*p* < 0.05) were recorded in levels of Ca and P_2_O_5_. The highest concentration of these species was observed following treatment of biochar and PA-800 without chemical fertilizer. Other work has demonstrated that biochar application increases Ca content in Komatsuna (*Brassica rapa* var. *perviridis*) [47], perhaps resulting from cations released from the biochar surface area into the soil solution. Increased P_2_O_5_ content in plant leaves may be attributed to high levels of organic acids in PA, which may solubilize soil P releasing usable phosphoric acid for plant uptake [16]. A beneficial interactive effect applying both biochar and PA was reported in other work [22]. The authors found a substantial micronutrient and inorganic nutrient supply, as well as slow-released active acids, and phenol components within PA. 

Concerning the effect of different input applications on soil physicochemical properties (Table 7), reduction of soil Na content following biochar application was observed to be significant (*p* < 0.05), and might be due to absorption of nutrients in the biochar surface. Biochar has a large surface area and high porosity, increasing absorption capacity in soil. This property may be advantageous, for example, in remediating soils with high salinity [48]. In terms of the effect of biochar in combination with PA at 800-fold and 200-fold dilutions (800 PA and 200 PA, respectively), significant reduction of soil nutrients Cu, Fe, and Mn was observed when compared to the sole PA treatments (800 PA and 200 PA). No significant difference was recorded in terms of pH when different treatments were applied (Table 7). Other researchers [13] have previously highlighted the importance of continuous PA application for remediation of soil pH. However, in this study, other detrimental effects were observed following PA application such as reduced soil enzymes and water holding capacity.

## 3. Materials and Methods

### 3.1. Pyrolysis Process

Biochar and PA materials were generated in the laboratory of Meiwa Co., Ltd. (Kanazawa, Japan). Biochar was generated with pyrolysis using a continuous-type rotary kiln (Carbon Hero, Kanazawa City, Ishikawa, Meiwa Co., Ltd., Japan). Wood chips of *Cryptomeria japonica* (Japanese cedar; Kidagen Lumber mill, Nomi, Japan) were used as kiln feedstock. Raw materials were dried and chopped before pyrolysis.

Pyrolysis temperature and duration was between 400 and 500 °C, for 20 to 30 min. Following pyrolysis, the biochar was sieved (2 mm) for homogeneity. Electroconductivity (EC) and pH of the biochar was 10.1 ms cm^−1^ and 3.2. Average surface area and porosity were 2.9052 × 10^2^ m^2^ g^−1^ and 0.68 nm, respectively. C, N, P, and K concentrations were 74.3%, 1.1%, 0.5%, and 1.7%, respectively. The C:N ratio was 67.5. Cation exchange capacity was 22 cmol(^+^) kg^−1^.

PA was also generated with pyrolysis using pine feedstock (*Pinus thunbergiana*); 40 kg of pine wood (moisture content of 10 to 20%) yielded approximately 3000 mL after 3 h under the same pyrolysis conditions used for biochar production. The sample was then analyzed with EEM, SEC-HPLC, CG-MS, and ion chromatography. 

### 3.2. Spectrometric Analysis and Chromatography

#### 3.2.1. Excitation Emission Matrix (EEM) Spectroscopy

A light path length of 10 mm (quartz cell, F10-SQF-10, GL Sciences, Tokyo, Japan) was used for EEM measurements. Fluorescence intensity was measured with a spectrophotometer (F07100, Hitachi, Tokyo, Japan), using a 5 nm interval. Excitation wavelength (Ex) and fluorescence wavelength (Em) ranged from 200–500 nm and 250–550 nm, respectively. Scan speed was between 30,000 nm min^−1^ and 60,000 nm min^−1^. The excitation and fluorescence slit was set to 5 nm and 10 nm, respectively. Voltage was 400 V. EEM equipment was allowed to settle for more than 1 h to stabilize the xenon lamp excitation source. A low temperature circulator (CTP-1000, EYELA, Queenstown, Singapore) was used to keep water temperature stable at 25 °C. MilliQ H_2_O and quinine sulfate were used as controls before and after measurement of the samples. Relative fluorescence intensity (RFI) was calculated based on quinine sulfate results, ranging between Ex/Em = 455 nm/350 nm. Measurements were replicated at least twice. Data analysis was performed with FL Solutions, version 4.2 (Hitachi, Tokyo, Japan). 

#### 3.2.2. Size Exclusion Chromatography—High-Performance Liquid Chromatography (SEC–HPLC)

Gel permeation chromatography (GPC) columns (GL-W530, 10.7 mm × 300 mm, Hitachi) with an exclusion limit of 50,000 Da were utilized, based on the method of Nagao et al. (2001). An HPLC system equipped with an L-2130 intelligent pump (Hitachi) allowed for SEC mobile phases, with a flow rate of 1.0 mL min^−1^ was used. A column oven allowed temperature to be fixed at 30 °C. An L-2485 (Hitachi) chromatography detector was used. A calibration curve was generated by analyzing blue dextran (50,000 Da), polyethylene glycol (C_2n_H_4n_+2O_n+1_), 1 Amino-2-hydroxymethyl-1,3-propanediol (C_4_H_11_NO_3_(0.01 M)), and NaCl (0.01 M) with a differential refractometer. pH was adjusted to 8.00 ± 0.03 with 1.0 M HCL. The machine was purged to remove bubbles and avoid salt precipitation, and the apparatus was left to settle for over 1 h; 1840, 6450, 1010, 400, 194, 106 Da, acetone (molecular size: 58 Da), MilliQ H_2_O, and fulvic acid (10 mg L^−1^) were used as controls of the HPLC system. Volume, peak area, and height were calculated with an HPLC system manager (D-7000, Hitachi). Measurements were replicated at least twice. Six peaks were observed with molecular weights of 8970, 5190, 1650, 790, 670, and 280 Da, inferred from a calibration curve generated using standard material.

#### 3.2.3. Ion Chromatography

Samples were diluted with ultrapure H_2_O, and purified to high levels of specification. Later, the samples were filtered through a 0.45 νm pore size filter (Merck KGaA, Darmstadt, Germany) before analysis. Organic acids (lactic acid, acetic acid, citric acid, malic acid, formic acid, and succinic acid) were analyzed using a Dionex ICS-2100 ion chromatography system and Ion Pac AS20 4 × 250 mm column (Thermo Fisher Scientific Inc., Waltham, MA, USA). Compounds were detected and quantified by measuring the magnitude of conductivity in the eluted fractions.

#### 3.2.4. Gas Chromatography-Mass Spectrometry (GC-MS)

An Agilent/JEOL gas chromatograph was used to identify organic components contained in the sample of the refined PA. A 30 m × 0.25 mm × 0.25 μm capillary column (Ultra ALLOY, Frontier Lab, Fukushima, Japan) was used. The injection volume and port temperature was 1.0 μL and 220 °C, respectively. Split injection was performed at a rate of 50:1. The carrier gas was helium, with a stable flow rate of 3.00 mL min^−1^. Column temperature was maintained at 40 °C for 2 min, then raised to 360 °C at a heating rate of 20 °C min^−1^, for 12 min. Electron impact (El) source energy was 70 eV, source temperature was 230 °C, and the scanning range was 35–400 amu s^−1^. The National Institute of Standards and Technology (NIST) mass spectrometry library was used for analysis. Corresponding peak areas were used to determine the relative compound content within PA samples. 

### 3.3. Field Trial

Komatsuna (*Brassica rapa* var. *perviridis*) (Takii & Co., Ltd., Kyoto, Japan) plants were cultivated in a randomized complete block design pot experiment. Plants were grown in a plastic greenhouse on an experimental farm at Meiwa Co. Ltd., Japan (36°37′24.7″ N, 136°37′58.7″ E). In July, Komatsuna seeds were sowed into plastic pots (0.038 m^2^) filled with 3.9 L of field soil. Three pots (9 plants per pot) were randomly selected for downstream experiments. The full experiment design is outlined in the Supplementary Materials (Table S1; Figure S1).

Chemical fertilizer and biochar were mixed into the soil at different rates. For the control pot (full fertilizer treatment, zero biochar, zero PA), the recommended rate of chemical fertilizer for Ishikawa prefecture was applied, equivalent to 2.13 g of 16:10:14 NPK fertilizer per pot. Within the treatment plots, 0, 50, 75, or 100% of inorganic fertilizer was replaced with biochar and/or PA. PA was diluted with water at two ratios: 1 part pyroligneous acid and 200 parts water (PA200), and 1 part pyroligneous acid and 800 parts water (PA800). After dilution, 200 mL of PA was mixed into the soil. To control for moisture content, 200 mL of water was supplied to the other treatments. pH and EC of the soil was 6.5 and 0.106 mS cm^−1^. Rates of chemical fertilizer, biochar, and PA applied to each control and treatment plot are shown in Table 1.

Ten days after sowing (DAS), seedlings were thinned to 4 plants per pot, and the remaining plants were harvested at 44 DAS. Temperature and humidity were monitored. All treatments were irrigated twice per day with 50 mL. After harvest, plants were gently washed and dried with tap water and paper towels. Plants were separated into two parts: leaves (above ground organs) and roots. Fresh weight was measured with a balance. Plant height was recorded as length from the base of the leaf stalk, to the tip of the longest leaf. Chlorophyll levels were recorded with a SPAD meter (Soil Plant Analysis: Development; SPAD-502, Konica Minolta, Osaka, Japan) at 23, 26, and 31 DAS. Chlorophyll levels were recorded in triplicate from the center of the smallest and largest leaf.

### 3.4. Fertility/Nutrition Analysis

#### 3.4.1. Soil

Soil sampling was performed at harvest. Soils were air-dried, ground, and passed through a 2 mm sieve prior to chemical analysis. pH was measured using the glass electrode method with a soil and water ratio of 1:2.5 [49]. To determine soil exchangeable cation capacity, extraction was performed with 1 M NH_4_OAc at pH 7 [50], then measured with a multitype inductive coupled plasma (ICP) emission spectrometer (ICPE-9000, Shimadzu Co, Kyoto, Japan). Concentrations of available micronutrients (Fe, Zn, Cu, and Mn) were measured by mixing 10 g of soil with 20 mL of diethylene triamine pentaacetic acid (DTPA-TEA) solution [51]. Available P was extracted with the Truog method quantified by using molybdenum blue [52].

#### 3.4.2. Plant

Above-ground organs were oven-dried at 60 °C, weighed for dry biomass, and homogenized in agate grinding jars with a mixer mill (MM200, Retsch GmbH, Haan, Germany); 0.5 g of the sample was digested with 1 mL HNO_3_ within Teflon vessels oven-heated to 160 °C for 4 h. Samples were left to rest overnight [53]. Concentrations of Ca, Mg, K, Na, P, Fe, Zn, Cu, and Mn were determined with a multitype ICP emission spectrometer (ICPE-9000, Shimadzu Co., Kyoto, Japan).

### 3.5. Statistical Analysis

All experiments were conducted in duplicate or triplicate. ANOVA tests (*p* < 0.05) were used to determine significant effects on plant height, fresh biomass, SPAD, and nutrient content in soil and plant tissues. The Shapiro–Wilk test was used to verify normality of the data. The three factors for the ANOVA tests were: PA dilution (200-fold, 800-fold, control); biochar level (0 g and 5 g); chemical fertilizer rate (0, 50, 75, and 100%). Statistical analyses were conducted with R (Rstudio 3.5.1 version, RStudio, Boston, MA, USA). Significant differences were verified at *p* < 0.05. 

## 4. Conclusions

Through different analyses of chemical composition, PA was shown to contain several compounds beneficial for plant growth, such as humic substances and amino acids for biostimulation, and creosol for anti-microbial properties. Our study evaluated the effectiveness of applying PA along with biochar in agricultural crop production. While PA is often considered an alternative pesticide, we examined its effect on other agriculturally important plant parameters. PA application at two dilution rates (200-fold and 800-fold) resulted in increased growth of Komatsuna plants, when applied without biochar and chemical fertilizer. Combined application of PA with biochar showed no effect on plant growth, but increased accumulation of leaf nutrients. Future studies are required to further explore physiological processes driving these effects (such as altered carbohydrate metabolism and secondary metabolism) for better understanding of the underlying mechanisms of PA on plant growth.

## Figures and Tables

**Table 1:** Different treatment combinations of pyroligneous acid (PA), biochar (B), and chemical fertilizer (CF). Percentages in the column of Biochar and Chemical Fertilizer represent proportions of these inputs applied within respective treatments.

Treatment Name	Pyroligneous Acid	Biochar	Chemical Fertilizer
Control	-	-	-
800PA	800-fold dilution	-	-
200PA	200-fold dilution	-	-
B	-	5%	-
800PA-B	800-fold dilution	5%	-
200PA-B	200-fold dilution	5%	-
50CF	-	-	50%
75CF	-	-	75%
100CF	-	-	100%
800PA-50CF	800-fold dilution	-	50%
800PA-75CF	800-fold dilution	-	75%
800PA-100CF	800-fold dilution	-	100%
200PA-50CF	200-fold dilution	-	50%
200PA-75CF	200-fold dilution	-	75%
200PA-100CF	200-fold dilution	-	100%
B-75CF	-	5%	75%
B-100CF	-	5%	100%
800PA-B-50CF	800-fold dilution	5%	50%
800PA-B-75CF	800-fold dilution	5%	75%
800PA-B-100CF	800-fold dilution	5%	100%
200PA-B-50CF	200-fold dilution	5%	50%
200PA-B-75CF	200-fold dilution	5%	75%
200PA-B-100CF	200-fold dilution	5%	100%

**Table 2:** Percentage (mass within pyroligneous acid, *m*/*v*) of detectable compounds in pyroligneous acid, analyzed by ion chromatography.

Name	Molecular Formula	Molecular Weight (g mol^−1^)	%
Acetic Acid	C_2_H_4_O_2_	60.05	73.86
Pyruvic acid	C_3_H_4_O_3_	88.06	23.42
Succinic acid	C_4_H_6_O_4_	118.09	1.98
Malic acid	C_4_H_6_O_5_	134.0874	0.74

**Table 3:** List of acid compounds in pyroligneous acid detected by gas chromatography-mass spectrometry (GC-MS), ordered by molar mass.

Name	Molecular Formula	Molecular Weight (g mol^−1^)
Acetic acid	C_2_H_4_O_2_	60.05
Cyclopentanone	C_5_H_8_O	84.12
2-Hydroxyethyl	C_4_H_8_O_3_	104.1
o-Guaiacol	C_7_H_8_O_2_	124.14
2-Methoxy-4-methylphenol	C_8_H_10_O_2_	138.16
Vanillin	C_8_H_8_O_3_	152.15
2′-hydroxy-5′-methoxyacetophenone	C_9_H_10_O_3_	166.17
levoglocosan	C_6_H_10_O_5_	162.141

**Table 4:** The effect of pyroligneous acid (PA) and biochar on fresh weight of Komatsuna (*Brassica rapa* var. *perviridis*). Values represent the means of three replicates. ×800 and ×200 represent PA dilution rates: 1 part PA, and either 800 or 200 parts water. Different lowercase letters indicate significant difference at *p* < 0.05 as indicated by Tukey’s tests. NS: not significant, **: *p*-value < 0.01, *: *p*-value < 0.05.

Pyroligneous AcidDilution Rate	Biochar Addition	Chemical Fertilizer Concentration			
		100%	75%	50%	0%
		Fresh Weight (g)			
Water (control)	No	19.9 ab	18.9 ab	18.9 a	9.4 bc
	Yes	20.0 ab	16.3 ab	-	9.0 bc
800	No	14.2 b	12.7 b	15.5 a	14.2 a
	Yes	19.8 ab	18.2 ab	15.8 a	7.5 c
200	No	18.8 ab	19.7 a	18.0 a	13.2 ab
	Yes	21.9 a	17.4 ab	13.2 a	8.4 c
ANOVA					*p* Value
	Pyroligneous acid (A)	NS			0.41
	Biochar (B)	*			0.02
	Chemical fertilizer (C)	**			<0.0001
	A × B	NS			0.09
	A × C	NS			0.15
	B × C	*			0.02
	A × B × C	*			0.02

**Table 5:** The effect of pyroligneous acid (PA) and biochar on plant height of Komatsuna (*Brassica rapa* var. *perviridis*). ×800 and ×200 represent PA dilution rates: 1 part PA, and either 800 or 200 parts water. Different lowercase letters indicate significant difference at *p* < 0.05. NS: not significant, **: *p*-value < 0.01, *: *p*-value < 0.05.

Pyroligneous AcidDilution Rate	Biochar Addition	Chemical Fertilizer Concentration			
		100%	75%	50%	0%
		Plant Height (cm)			
Water (control)	No	22.4 a	22.0 a	22.3 a	17.8 abc
	Yes	22.2 a	20.0 a	-	16.5 bc
800	No	19.3 b	20.0 a	22.0 a	20.3 a
	Yes	22.4 a	21.4 a	21.9 a	15.6 c
200	No	21.8 a	22.3 a	21.8 a	19.0 ab
	Yes	23.2 a	21.0 a	20.5 a	16.4 bc
ANOVA					*p* Value
	Pyroligneous acid (A)	NS			0.40
	Biochar (B)	**			0.01
	Chemical fertilizer (C)	**			<0.0001
	A × B	*			0.04
	A × C	NS			0.06
	B × C	*			0.02
	A × B × C	**			0.01

**Table 6:** Nutrient content in Komatsuna plant leaf samples following application of different treatments. 800PA: pyroligneous acid diluted 800-fold; 200PA: pyroligneous acid diluted 200-fold; B: Biochar; 50CF: 50% chemical fertilizer; 75CF: 75% chemical fertilizer; 100CF: 100% chemical fertilizer. Different lowercase letters indicate significant difference at *p* < 0.05 as indicated by Tukey’s tests.

Treatment Name	Ca (mg/kg)	Cu (mg/kg)	Fe (mg/kg)	K (mg/kg)	Mg (mg/kg)	Mn (mg/kg)	Na (mg/kg)	P_2_O_5_ (mg/kg)	Zn (mg/kg)
Control	25,066.67 ab	23.97 ab	811.33 a	37,066.67 a	6690 a	145.7 ab	1826.67 a	14,366.67 ab	322.33 a
800PA	20,966.67 ab	17.73 ab	611.67 a	25,300 a	4846.67 ab	91.27 ab	1381 a	11,266.67 ab	216.33 a
200PA	22,900 ab	18.47 ab	727 a	30,300 a	5980 ab	200.67 a	1302.33 a	14,866.67 ab	294.33 a
B	22,300 ab	16.57 ab	670 a	25,433.33 a	5566.67 ab	78.5 ab	1407.33 a	13,360 ab	218.33 a
800PA-B	27,100 a	17.93 ab	410.33 a	30,366.67 a	6410 ab	107.1 ab	1396.67 a	20,666.67 a	265.33 a
200PA-B	24,166.67 ab	17.9 ab	341.67 a	26,833.33 a	6373.33 ab	93.03 ab	1063.33 a	18,466.67 ab	282.67 a
50CF	15,833.33 ab	11.77 b	229.33 a	24,500 a	4156.67 ab	114.6 ab	1193.33 a	9683.33 ab	137.67 a
75CF	20,700 ab	14.67 b	710.67 a	29,300 a	5500 ab	174.33 ab	1466.67 a	14,666.67 ab	273.67 a
100CF	18,333.33 ab	19.37 ab	316.67 a	30,966.67 a	4816.67 ab	120 ab	1623.33 a	9263.33 b	181 a
800PA-50CF	14,633.33 b	15.5 ab	133 a	30,600 a	3580 b	59.8 b	1303.33 a	8560 b	103.67 a
800PA-75CF	19,900 ab	60.9 a	209 a	21,633.33 a	4080 ab	66.77 b	866.67 a	11,416.67 ab	201.67 a
800PA-100CF	16,966.67 ab	19.9 ab	244.67 a	36,033.33 a	4100 ab	100.4 ab	1566.67 a	11,630 ab	161.67 a
200PA-50CF	17,533.33 ab	17.3 ab	261 a	24,866.67 a	4480 ab	82.03 ab	1506.67 a	9186.67 b	124.7 a
200PA-75CF	22,633.33 ab	20.97 ab	609.67 a	28,233.33 a	5483.33 ab	109.8 ab	1406.67 a	11,380 ab	195.33 a
200PA-100CF	24,666.67 ab	22.57 ab	195.33 a	26,033.33 a	5783.33 ab	67.8 b	1723.33 a	14,066.67 ab	141 a
B-75CF	23,566.67 ab	22.4 ab	328.33 a	34,566.67 a	5903.33 ab	128.4 ab	1530 a	11,383.33 ab	231.67 a
B-100CF	20,933.33 ab	20.3 ab	237 a	29,866.67 a	5050 ab	73.03 ab	1456.67 a	10,183.33 ab	167.67 a
800PA-B-50CF	26,300 a	20.67 ab	222.67 a	32,633.33 a	6066.67 ab	111.8 ab	1553.33 a	13,700 ab	227.67 a
800PA-B-75CF	18,800 ab	17.1 ab	275 a	32,333.33 a	4663.33 ab	69.83 b	1496.67 a	15,403.33 ab	164 a
800PA-B-100CF	16,900 ab	15.73 ab	165 a	19,366.67 a	4003.33 ab	89.87 ab	1190 a	11,706.67 ab	168.67 a
200PA-B-50CF	22,733.33 ab	20.57 ab	246.33 a	35,300 a	5543.33 ab	92.5 ab	1516.67 a	12,433.33 ab	187.67 a
200PA-B-75CF	16,766.67 ab	16.47 ab	192.33 a	22,300 a	4300 ab	115.03 ab	1006 a	10,680 ab	215.67 a
200PA-B-100CF	22,066.67 ab	22.63 ab	265.67 a	40,066.67 a	5696.67 ab	123.53 ab	1573.33 a	17,200 ab	249.33 a

**Table 7:** Soil properties/nutrients of Komatsuna pots following application of different treatments. 800PA: pyroligneous acid diluted 800-fold; 200PA: pyroligneous acid diluted 200-fold; B: Biochar; 50CF: 50% chemical fertilizer; 75CF: 75% chemical fertilizer; 100CF: 100% chemical fertilizer. Different lowercase letters indicate significant difference at *p* < 0.05 as indicated by Tukey’s tests.

Treatment Name	pH	EC	Phosphate	DTPA Extracted Available Micronutrient (mg/kg)							
	(H_2_O)	(mS/cm)	(mgP_2_O_5_/kg)	Cu	Fe	Mn	Zn	Ca	K	Mg	Na
Control	5.82 abc	18.51 ab	3095 a	3.43 a	159 abcde	12.2 abc	16.7 c	21.33 a	1 abcde	9.3 a	0.5 a
800PA	5.67 abc	18.75 ab	3069 a	2.97 abcde	149.67 abcdef	14.63 abc	12.5 c	19.23 abc	0.97 abcdef	7.87 bc	0.4 abc
200PA	5.61 bc	12.65 ab	2884 a	3.13 abcde	160.67 abcde	13.87 abc	14.1 c	20.6 ab	0.9 cdefg	8.47 ab	0.47 ab
B	5.76 abc	18.32 ab	3025 a	3.13 abcde	166.33 abcd	16.37 ab	13.33 c	19.4 abc	1.07 abc	7.87 bc	0.4 abc
800PA-B	5.82 abc	10.3 ab	3143 a	2.7 cde	126.33 def	8.47 c	12.27 c	18.6 bc	1.03 abcd	7.63 bc	0.4 abc
200PA-B	5.8 abc	16.71 ab	2734 a	2.73 bcde	137.67 bcdef	9.03 c	11.47 c	18.73 bc	1.03 abcd	7.87 bc	0.4 abc
50CF	5.56 c	12.24 ab	2901 a	2.9 abcde	137.33 bcdef	11.33 abc	13.67 c	19.2 abc	1.1 ab	8.13 bc	0.4 abc
75CF	5.77 abc	17.49 ab	3087 a	3.1 abcde	149.33 abcdef	16.7 ab	15.2 c	17.9 c	0.97 abcdef	7.87 bc	0.37 abc
100CF	5.65 bc	20.23 ab	3173 a	3.2 abcd	156.33 abcdef	14.73 abc	15.4 c	19.5 abc	0.77 g	7.9 bc	0.37 abc
800PA-50CF	5.79 abc	17.92 ab	3055 a	2.57 de	120.67 ef	11.3 abc	13.37 c	19.8 abc	0.9 cdefg	7.6 bc	0.3 c
800PA-75CF	5.95 a	11.27 ab	2981 a	2.5 e	113 f	8.8 c	12.67 c	20.1 abc	0.93 bcdefg	7.6 bc	0.33 bc
800PA-100CF	5.6 bc	16.02 ab	3289 a	3.17 abcde	178.67 ab	13.57 abc	16.9 c	19.53 abc	0.97 abcdef	7.43 c	0.37 abc
200PA-50CF	5.74 abc	10.62 ab	3027 a	3.03 abcde	150.33 abcdef	14.43 abc	12.4 c	20.1 abc	0.97 abcdef	7.9 bc	0.3 c
200PA-75CF	5.87 ab	14.77 ab	3014 a	3.43 a	158.33 abcde	11.9 abc	15.5 c	20.5 ab	0.9 cdefg	7.67 bc	0.33 bc
200PA-100CF	5.94 a	8.48 b	3081 a	2.7 cde	132.67 cdef	10.4 bc	14.33 c	20.47 ab	0.87 defg	7.6 bc	0.33 bc
B-75CF	5.68 abc	17.11 ab	3156 a	3.4 ab	193.67 a	15.43 abc	16.27 c	19.03 bc	1 abcde	7.3 c	0.3 c
B-100CF	5.87 ab	16.11 ab	3406 a	3.2 abcd	164.33 abcde	11.23 abc	18.8 c	19.97 abc	1.13 a	7.67 bc	0.37 abc
800PA-B-50CF	5.7 abc	15.34 ab	3031 a	3.1 abcde	170 abcd	12.37 abc	50.87 a	18.77 bc	0.97 abcdef	7.67 bc	0.33 bc
800PA-B-75CF	5.71 abc	28.1 a	3121 a	3.17 abcde	153 abcdef	9.57 bc	49.33 ab	19.07 bc	0.9 cdefg	7.53 c	0.4 abc
800PA-B-100CF	5.79 abc	13.02 ab	3243 a	3.2 abcd	160 abcde	13.73 abc	37.27 b	19.07 bc	0.8 fg	7.43 c	0.33 bc
200PA-B-50CF	5.84 abc	10.67 ab	3237 a	3.27 abc	170.33 abcd	11.23 abc	18.07 c	18.77 bc	1.03 abcd	7.57 bc	0.3 c
200PA-B-75CF	5.73 abc	12.35 ab	3281 a	3.23 abcd	175.33 abc	17.83 a	48.77 ab	19.3 abc	0.83 efg	7.97 bc	0.33 bc
200PA-B-100CF	5.62 bc	17.35 ab	3154 a	3.13 abcde	178 ab	17.6 a	37.8 b	19 bc	0.83 efg	8.47 ab	0.4 abc

## Data Availability

Data is contained within the article or Supplementary Materials.

## Supplementary Materials

The following supporting information can be downloaded at: https://www.mdpi.com/article/10.3390/molecules27113397/s1. Table S1: Randomized complete block design of pot experiment with three replicates across Block A (blue), Block B (Yellow), and Block C (Green). 100CF: 100% chemical fertilizer application; 75CF: 75% chemical fertilizer application; 50CF: 50% chemical fertilizer application; B: Biochar application; 800PA: Pyroligneous acid (800-fold dilution) application; 200PA: Pyroligneous acid (200-fold dilution) application; Figure S1: Picture of randomized complete block design pot experiment.

## Sample Availability

Samples of the compounds are not available from the authors.

## References

[1] Aguirre J.L., Baena J., Martín M.T., Nozal L., González S., Manjón J.L., Peinado M. (2020). Composition, Ageing and Herbicidal Properties of Wood Vinegar Obtained through Fast Biomass Pyrolysis. Energies.

[2] Cheng J., Hu S.C., Kang K., Li X.M., Geng Z.C., Zhu M.Q. (2021). The effects of pyrolysis temperature and storage time on the compositions and properties of the pyroligneous acids generated from cotton stalk based on a polygeneration process. Ind. Crops Prod..

[3] Medeiro L.C.D., Gaparotto L.C.S. (2022). Pyroligneous acid and antibacterial activity: Criticism of a paper by Araújo et al. (2018). J. Appl. Microbiol..

[4] de Souza Araújo E., Pimenta A.S., Feijó F.M.C., Castro R.V.O., Fasciotti M., Monteiro T.V.C., de Lima K.M.G. (2018). Antibacterial and antifungal activities of pyroligneous acid from wood of *Eucalyptus urograndis* and *Mimosa tenuiflora*. J. Appl. Microbiol..

[5] Guo G., Wang Q., Huang Q., Fu Q., Liu Y., Wang J., Hu S., Mašek O., Wang L., Zhang J. (2021). Effect of pyrolysis temperature on the characterisation of dissolved organic matter from pyroligneous acid. Molecules.

[6] Wang H.F., Wang J.L., Wang C., Zhang W.Z., Liu J.X., Dai B. (2012). Effect of bamboo vinegar as an antibiotic alternative on growth performance and fecal bacterial communities of weaned piglets. Livest. Sci..

[7] da Silva Porto F.G., Campos Â.D., Garcia I.T.S. (2019). Distilled pyroligneous liquor obtained from Eucalyptus grandis and chitosan: Physicochemical properties of the solution and films. Environ. Sci. Pollut. Res..

[8] Almeida R.S.R., Taccini M.M., de Moura L.F., Ceribelli U.L., Brito J.O., Gloria E.M. (2019). Potential of pyroligneous extract of Eucalyptus wood as a preservative of cosmetic and sanitizing products. Waste Biomass Valorization.

[9] Mohammadi-Aragh M.K., Stokes C.E., Street J.T., Linhoss J.E. (2021). Effects of loblolly pine biochar and wood vinegar on poultry litter nutrients and microbial abundance. Animals.

[10] Hua D., Fan Q., Zhao Y., Xu H., Chen L., Si H., Li Y. (2020). Continuous anaerobic digestion of wood vinegar wastewater from pyrolysis: Microbial diversity and functional genes prediction. Front. Bioeng. Biotechnol..

[11] Mathew S., Zakaria Z.A., Musa N.F. (2015). Antioxidant property and chemical profile of pyroligneous acid from pineapple plant waste bio-mass. Process Biochem..

[12] Nunkaew T., Kantachote D., Chaiprapat S., Nitoda T., Kanzaki H. (2018). Use of wood vinegar to enhance 5-aminolevulinic acid production by selected *Rhodopseudomonas palustris* in rubber sheet wastewater for agricultural use. Saudi J. Biol. Sci..

[13] Maliang H., Tang L., Lin H., Chen A., Ma J. (2020). Influence of high-dose continuous applications of pyroligneous acids on soil health assessed based on pH, moisture content and three hydrolases. Environ. Sci. Pollut. Res. Int..

[14] Lievens C., Mourant D., Hu X., Wang Z.Y., Wu L., Rossiter A., Gunawan R., He M., Li C.Z. (2018). A case study: What is leached from mallee biochars as a function of pH?. Environ. Monit. Assess..

[15] Zhu K., Gu S., Liu J., Luo T., Khan Z., Zhang K., Hu L. (2021). Wood vinegar as a complex growth regulator promotes the growth, yield, and quality of rapeseed. Agronomy.

[16] Grewal A., Abbey L., Gunupuru L.R. (2018). Production, prospects and potential application of pyroligneous acid in agriculture. J. Anal. Appl. Pyrolysis.

[17] Wang Y., Qiu L., Song Q., Wang S., Wang Y., Ge Y. (2019). Root proteomics reveals the effects of wood vinegar on wheat growth and subsequent tolerance to drought stress. Int. J. Mol. Sci..

[18] Jindo K., Canellas L.P., Albacete A., Figueiredo dos Santos L., Frinhani Rocha R.L., Carvalho Baia D., Oliveira Aguiar Canellas N., Goron T.L., Olivares F.L. (2020). Interaction between humic substances and plant hormones for phosphorous acquisition. Agronomy.

[19] Hua D., Fan O., Zhao Y., Xu H., Chen L., Li Y. (2020). Comparison of methanogenic potential of wood vinegar with gradient loads in batch and continuous anaerobic digestion and microbial community analysis. Sci. Total Environ..

[20] Sharchami T., Batta N., Berruti F. (2021). Production and separation of acetic acid from pyrolysis oil of lignocellulosic biomass: A review. Biofuels Bioprod. Biorefin..

[21] Zulkarami B., Ashrafuzzaman M., Husni M.O., Ismail M.R. (2011). Effect of pyroligneous acid on growth, yield and quality improvement of rockmelon in soilless culture. Aust. J. Crop Sci..

[22] Luo X., Wang Z., Meki K., Wang X., Liu B., Zheng H., You X., Li F. (2019). Effect of co-application of wood vinegar and biochar on seed germination and seedling growth. J. Soils Sediments.

[23] Shen R., Zhao L., Yao Z., Feng J., Jing Y., Watson J. (2020). Efficient Treatment of Wood Vinegar via Microbial Electrolysis Cell With the Anode of Different Pyrolysis Biochars. Front. Energy Res..

[24] Hagner M., Penttinen M.O., Tiilikkala K., Setälä H. (2013). The effects of biochar, wood vinegar and plants on glyphosate leaching and degradation. Eur. J. Soil Biol..

[25] Pan X., Zhang Y., Wang X., Liu G. (2017). Effect of adding biochar with wood vinegar on the growth of cucumber. IOP Conf. Ser. Earth Environ. Sci..

[26] Pimenta A.S., Fasciotti M., Monteiro T.V.C., Lima K.M.G. (2018). Chemical Composition of Pyroligneous Acid Obtained from Eucalyptus GG100 Clone. Molecules.

[27] Suresh G., Pakdel H., Rouissi T., Brar S.K., Fliss I., Roy C. (2019). In vitro evaluation of antimicrobial efficacy of pyroligneous acid from softwood mixture. Biotechnol. Res. Innov..

[28] Ruscalleda M., Seredynska-Sobecka B., Ni B.J., Arvin E., Balaguer M.D., Colprim J., Smets B.F. (2014). Spectrometric characterization of the effluent dissolved organic matter from an anammox reactor shows correlation between the EEM signature and anammox growth. Chemosphere.

[29] Tacoone M.I., Fernández R.A., Molina F.L., Gustín I., Sánchez C.G., Dassie S.A., Pino G.A. (2020). On the photophysics of electrochemically generated silver nanoclusters: Spectroscopic and theoretical characterization. Phys. Chem. Chem. Phys..

[30] Han F., Wei D., Ngo H.H., Guo W., Xu W., Du B., Wei Q. (2018). Performance, microbial community and fluorescent characteristic of microbial products in a solid-phase denitrification biofilm reactor for WWTP effluent treatment. J. Environ. Manag..

[31] Xu J., Luo H.W., Wang Y.K., Sheng G.P. (2015). Fluorescence approach for investigating binding properties between metals and soluble microbial products from a biological wastewater treatment plant. Process Biochem..

[32] Zhang Y., Liu X., Osburn C.L., Wang M., Qin B., Zhou Y. (2013). Photobleaching Response of Different Sources of Chromophoric Dissolved Organic Matter Exposed to Natural Solar Radiation Using Absorption and Excita-tion–Emission Matrix Spectra. PLoS ONE.

[33] Mostofa M.G.K., Liu C.Q., Yoshioka T., Vione D., Zhang Y., Sakugawa H. (2012). Fluorescent dissolved organic matter in natural waters. Photobiogeochemistry of Organic Matter, Environmental Science and Engineering.

[34] Gondek K., MierzwaHersztek M. (2021). Effect of Soil-Applied L-tryptophan on the Amount of Biomass and Nitrogen and Sulfur Utilization by Maize. Agronomy.

[35] Casadesús A., Pérez-Llorca M., Munné-Bosch S., Polo J. (2020). An enzymatically hydrolyzed animal protein-based biostimulant (pepton) increases salicylic acid and promotes growth of tomato roots under Temperature and nutrient stress. Front. Plant Sci..

[36] Canellas L.P., Olivares F.L., Okorokova-Façanha A.L., Façanha A.R. (2002). Humic acids isolated from earthworm compost enhance root elongation, lateral root emergence, and plasma membrane H+-ATPase activity in maize roots. Plant Physiol..

[37] Nagao S., Matsunaga T., Suzuki Y., Ueno T., Amano H. (2003). Characteristics of humic substances in the Kuji River waters as determined by high-performance size exclusion chromatography with fluorescence detection. Water Res..

[38] Nagao S., Baiting Y., Kim V.I., Shesterkin V.P., Leveshina S.I., Yoh M., Suzuki T., Kodama H., Terashima M., Seki O., Haruyama S., Shiraiwa T. (2015). Water chemistry of the middle Amur River. Chapter 5, In Environmental Change and the Social Response in the Amur River Baso, International Perspectives in Geography 5.

[39] Suzuki T., Nagao S., Horiuchi M., Maie N., Yamamoto M., Nakamura K. (2015). Characteristics and behavior of dissolved organic matter in the Kumaki River, Noto Peninsula, Japan. Limnology.

[40] Canellas L.P., Olivares F.L., Aguiar N.O., Jones D.L., Nebbioso A., Mazzei P., Piccolo A. (2015). Humic and fulvic acids as biostimulants in horticulture. Sci. Hortic..

[41] Capstaff N.M., Morrison F., Cheema J., Brett P., Hill L., Muñoz-García J.C., Khimyak Y.Z., Domoney C., Miller A.J. (2020). Fulvic acid increases forage legume growth inducing preferential up-regulation of nodulation and signalling-related genes. J. Exp. Bot..

[42] Rao H., Li P., Wu H., Liu C., Peng W., Su W. (2019). Simultaneous determination of six compounds in destructive distillation extracts of hawthorn seed by GC-MS and evaluation of their antimicrobial activity. Simultaneous determination of six compounds in destructive distillation extracts of hawthorn seed by GC-MS and evaluation of their antimicrobial activity. Molecules.

[43] Oramahi H.A., Yoshimura T., Diba F., Setyawati D., Nurhaida (2018). Antifungal and antitermitic activities of wood vinegar from oil palm trunk. J. Wood Sci..

[44] Yang J.F., Yang C.H., Liang M.T., Gao Z.J., Wu Y.W., Chuang L.Y. (2016). Chemical Composition, Antioxidant, and Antibacterial Activity of Wood Vinegar from Litchi chinensis. Molecules.

[45] Setiawati E., Annisa W., Soedarmanto H., Iskandar T. (2019). Characterization of neutralized wood vinegar derived from durian wood (Durio zibethinus) and its prospect as pesticide in acidic soil. IOP Conf. Ser. Earth Environ. Sci..

[46] Chen J., Wu J.H., Si H.P., Lin K.Y. (2016). Effects of adding wood vinegar to nutrient solution on the growth, photosyntehsis and absorption of mineral elements of hydropnoic lettuce. J. Plant Nutr..

[47] Basalirwa D., Sudo S., Wacal C., Oo A.Z., Sasagawa D., Yamamoto S., Masunaga T., Nishihara N. (2020). Impact of fresh and aged palm shell biochar on N_2_O emissions, soil properties, nutrient content and yield of Komatsuna (Brassica rapa var. perviridis) under sandy soil conditions. Soil Sci. Plant Nutr..

[48] Akhtar S.S., Andersen M.N., Liu F. (2015). Biochar mitigates salinity stress in potato. J. Agron. Crop Sci..

[49] Thunjai T., Boyd C.E., Dube K. (2001). Pond soil pH measurement. J. World Aquac. Soc..

[50] Thomas G.W., Page A.L. (1982). Exchangeable Cations. Methods Soil Analysis. Part 2, Agronomy Monographs 9.

[51] Lindsay W.L., Norvell W.A. (1978). Development of a DTPA soil test for zinc, iron, manganese, and copper. Soil Sci. Soc. Am. J..

[52] Truog E. (1930). The determination of readily available phosphorus in soils. J. Am. Soc. Agron..

[53] Koyama T., Sutoh M. (1987). Simultaneous multi element determination of soils, plant and animal samples by inductively coupled plasma emission spectrophotometry. Jpn. J. Soil Sci. Plant Nutr..

