# Supplementary material for: Sustainable Plant Growth Promotion and Chemical Composition of Pyroligneous Acid When Applied with Biochar as a Soil Amendment
Source: Molecules. 2022 May 25;27(11):3397. doi: 10.3390/molecules27113397 (PMC9182051; doi:10.3390/molecules27113397)
Supplement: Supplementary file 1 [file molecules-27-03397-s001.zip › molecules-1714093-SI.pdf]

Supplementary material

Supplementary material Table S1: Randomized complete block design of pot experiment with three replicates across Block A (blue), Block B (Yellow), and Block C (Green). 100CF: 100% chemical fertilizer application; 75CF: 75% chemical fertilizer application; 50CF: 50% chemical fertilizer application; B: Biochar application; 800PA: Pyroligneous acid (800-fold dilution) application; 200PA: Pyroligneous acid (200-fold dilution) application.

100CF	Control	200PA-50CF	800PA-75CF	200PA-75CF	200PA-50CF	200PA	800PA-50CF	100CF	800PA-100CF	50CF	200PA	Reference Soil	B-75CF	200PA-100CF	800PA-50CF	B	75CF
B	200PA-100CF	200PA-75CF	200PA	800PA-100CF	800PA	200PA-100CF	200PA-75CF	Control	800PA75CF	800PA	PA-B-75CF	200PA-75CF	200PA-50CF	800PA-B-100CF	PA-B-75CF	800PA	200PA-B-100CF
B-75CF	800PA-50CF	75CF	800PA-50CF	Reference Soil	200PA-B-100CF	800PA	Reference Soil	75CF	800PA-100CF	800PA-50CF	B	200PA-50CF	800PA-100CF	Control	200PA	200PA-75CF	800PA-B
800PA-B-75CF	50CF	800PA	800PA-B-100CF	B-100CF	200PA	200PA-100CF	200PA-75CF	B-100CF	200PA-50CF	200PA-50CF	B-75CF	200PA	B-100CF	800PA-50CF	100CF	800PA-75CF	50CF

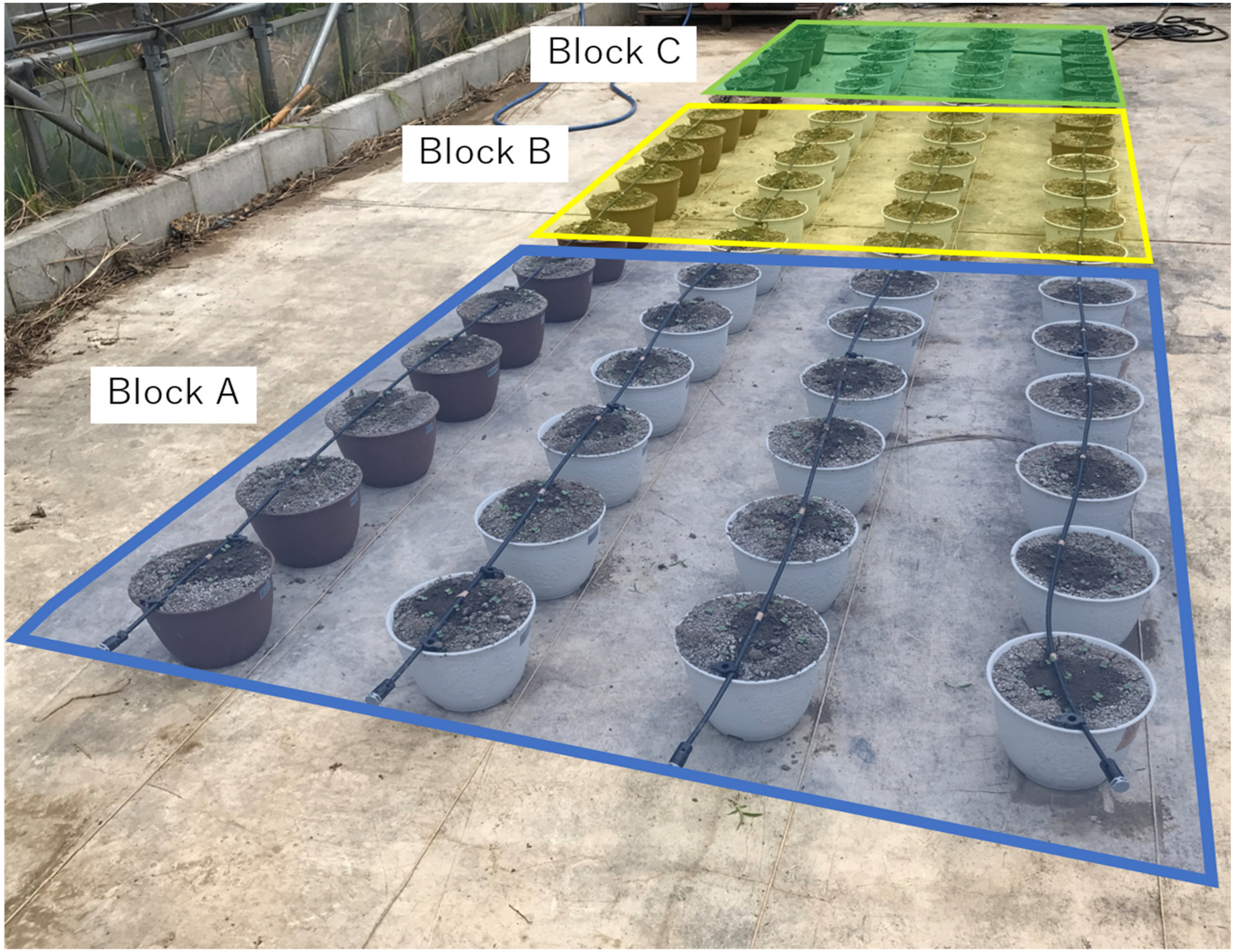

Supplementary material Figure S1: Picture of randomized complete block design pot experiment.
